# Advances in the Application of Chemical Peels in the Treatment of Pigmentary Skin Disorders

**DOI:** 10.1111/jocd.71048

**Published:** 2026-07-10

**Authors:** Yuxin Li, Wenzhong Xiang

**Affiliations:** ^1^ Department of Dermatology Zhejiang Chinese Medical University Hangzhou Zhejiang China; ^2^ Department of Dermatology Hangzhou Third People's Hospital Hangzhou Zhejiang China

**Keywords:** chemical peel, glycolic acid, melasma, postinflammatory hyperpigmentation, salicylic acid, solar lentigines, trichloroacetic acid

## Abstract

**Background:**

Pigmentary disorders, including melasma, postinflammatory hyperpigmentation (PIH), solar lentigines, Riehl's melanosis, and periorbital hyperpigmentation, are common concerns in cosmetic dermatology, particularly in patients with skin of color. Chemical peels remain widely used due to their accessibility, flexibility, and compatibility with combination therapies.

**Aims:**

To summarize current clinical evidence on chemical peels for common pigmentary skin disorders and highlight practical considerations for cosmetic dermatology.

**Methods:**

PubMed and citation tracking were used to identify representative English‐language studies and reviews published up to January 2026. Emphasis was placed on randomized trials, split‐face studies, prospective cohorts, case series, and recent reviews addressing peel agents, protocols, efficacy, and safety.

**Results:**

Superficial glycolic acid is supported by the strongest evidence, especially for melasma, where it performs best as an adjunct to photoprotection and depigmenting therapy. Salicylic acid is particularly useful for acne‐prone patients and PIH because of its keratolytic and anti‐inflammatory properties. Trichloroacetic acid can be effective for selected localized lesions and some melasma protocols but has a narrower safety margin in darker phototypes. Evidence for Jessner's solution, lactic acid, retinoid peels, and peels used for Riehl's melanosis, solar lentigines, and periorbital hyperpigmentation is encouraging but less standardized. Across disorders, outcomes depend on cautious depth selection, priming, interval control, and maintenance therapy.

**Conclusions:**

Chemical peels are best used as individualized adjuncts rather than stand‐alone treatments for pigmentary disease. In routine cosmetic practice, superficial glycolic acid and salicylic acid peels offer the most consistent balance of efficacy, tolerability, and versatility.

## 1 Introduction

Pigmentary skin disorders are common reasons for consultation in cosmetic dermatology. Although benign, they are often chronic, highly visible, and psychologically burdensome. Management is especially challenging in Fitzpatrick skin types III–VI, in whom inflammation from either disease or treatment can exacerbate dyspigmentation [1, 2]. Conventional therapies such as sunscreen, hydroquinone, azelaic acid, retinoids, triple‐combination cream, and oral or device‐based treatments remain central, but relapse and incomplete clearance are common [3]. Chemical peels induce controlled epidermal injury, promoting desquamation, melanin dispersion, and enhanced penetration of topical agents [4]. Compared with lasers, peels are relatively inexpensive, technically accessible, and repeatable [5]. However, their benefit depends on careful selection of peeling agent, concentration, contact time, priming, and postprocedure care. In darker skin, overly aggressive treatment may worsen PIH [6, 7]. This review summarizes clinical evidence and practical considerations for chemical peels in pigmentary disorders.

## 2 Methods

Literature was identified mainly through PubMed and citation tracking using combinations of terms related to peeling agents (“chemical peel,” “glycolic acid,” “salicylic acid,” “trichloroacetic acid,” “Jessner,” “lactic acid,” “retinoid peel”) and pigmentary disorders (“melasma,” “postinflammatory hyperpigmentation,” “solar lentigines,” “Riehl melanosis,” “pigmented contact dermatitis,” “periorbital hyperpigmentation”). English‐language randomized trials, split‐face studies, prospective studies, retrospective series, case reports, and narrative or systematic reviews were screened.

Studies reporting clinical outcomes, protocols, tolerability, adverse effects, or recurrence were prioritized, especially comparative trials, contemporary reviews, and studies involving darker phototypes.

## 3 Applications of Chemical Peels in Different Pigmentary Disorders

### 3.1 Melasma

Melasma has the strongest evidence base among pigmentary indications for chemical peeling. It is a chronic acquired disorder influenced by ultraviolet and visible light, hormonal factors, genetic predisposition, inflammation, and vascular and basement membrane changes [8, 9]. Given the high recurrence rate, chemical peels are typically used as adjuncts rather than monotherapy.

Glycolic acid (GA) has become one of the most widely used chemical peeling agents in clinical practice. Its small molecular size, predictable penetration, and wide clinical familiarity make it attractive for repeated superficial treatment [4, 10]. Across split‐face and comparative studies, serial GA peels improve melasma severity scores and patient‐rated pigmentation, particularly in epidermal or mixed melasma [11, 12]. Several reports found GA comparable or superior to trichloroacetic acid (TCA), retinoid peels, or alternative superficial peels, often with fewer adverse effects and better patient acceptance [13, 14, 15, 16]. In practice, GA is most effective when introduced gradually and combined with topical therapy.

Lactic acid has also shown benefit in melasma and may be useful in sensitive skin because of its humectant properties, but the supporting literature is smaller and protocols vary [10, 17]. Jessner's solution and retinoid‐based peels have likewise shown benefit in some cohorts, but they are less standardized and often studied within combination regimens, making direct comparisons difficult [18, 19, 20].

TCA occupies a more selective place in melasma management. At low concentrations it can improve epidermal pigment and may be useful in resistant focal areas or within carefully supervised protocols [14]. However, its therapeutic window is narrower than that of GA. Burning, frosting, prolonged erythema, rebound hyperpigmentation, and delayed dyspigmentation occur more readily when concentration or preparation is not carefully matched to phototype and barrier status [15]. For this reason, many clinicians reserve TCA for selected patients, smaller treatment fields, or situations in which they have substantial procedural experience.

Salicylic acid (SA) is another useful option, especially in patients with oily skin or coexisting acne. Beyond its keratolytic action, SA has anti‐inflammatory and comedolytic properties [15]. Conventional and supramolecular formulations have both shown improvement in melasma, although the evidence base remains smaller than for GA [21]. SA is often tolerated well in serial superficial treatment, but irritation still occurs when the barrier is impaired or when it is layered too aggressively with other active topicals.

Combination therapy consistently outperforms monotherapy in melasma. Studies combining GA peels with azelaic acid, hydroquinone‐containing regimens, oral tranexamic acid, microdermabrasion, or selected light‐based procedures generally report faster or greater pigment reduction than a single modality alone [12, 13, 16, 22, 23]. However, increased efficacy can come at the cost of more irritation and more complex aftercare. Overall, superficial GA remains the most practical first‐line peel for melasma, while SA, lactic acid, Jessner's solution, and carefully selected TCA protocols serve as alternatives or adjuncts.

An important practical point is that not all melasma responds equally. Epidermal disease usually improves more readily than dermal or long‐standing mixed melasma, and patients with frequent relapse often require maintenance cycles rather than a single intensive course. Pretreatment with hydroquinone or other lightening agents may reduce uneven penetration and help suppress rebound melanogenesis after each session. Conversely, patients with very sensitive skin, active dermatitis, or barrier dysfunction may need a simplified regimen before peeling is attempted.

Protocol design also matters. Even with the same named peeling agent, outcomes can differ according to concentration, number of coats, contact time, interval between sessions, and whether neutralization is required. These variables likely explain part of the inconsistency between studies. In practice, starting with lower‐intensity, serial treatments is often preferable to pursuing faster clearance with a more aggressive first procedure, especially in skin of color.

The clinical characteristics of commonly used chemical peeling agents for melasma are summarized in Table 1.

**TABLE 1:** Clinical characteristics of commonly used chemical peeling agents for melasma.

Agent	Typical concentration/depth	Main mechanism	Suitable type	Safety notes
Glycolic acid (GA)	30%–70%; very superficial to superficial	Accelerates epidermal turnover; removes melanin‐laden corneocytes; enhances topical penetration [4, 10]	Epidermal; mixed	Transient stinging, burning, erythema, and scaling are common; higher concentrations and combined procedures require cautious escalation [4, 13, 24]
Trichloroacetic acid (TCA)	10%–35%; superficial to medium‐depth	Protein coagulation followed by epidermal regeneration and superficial remodeling [25]	Epidermal; mixed; selected resistant focal disease	Narrower safety margin, especially in darker phototypes; excessive depth increases PIH, hypopigmentation, scarring, and rebound dyschromia [15, 25]
Salicylic acid (SA)	20%–30%; superficial	Lipophilic keratolysis; anti‐inflammatory and comedolytic effects; reduces epidermal pigment retention [26]	Epidermal; acne‐prone or oily skin; adjunctive use	Usually well tolerated, but barrier impairment or aggressive layering can cause irritation and secondary PIH [21, 24]
All‐trans retinoic acid (ATRA)/retinoid peel	Topical 0.05%–0.1%; peel about 1%	Promotes epidermal turnover; inhibits melanogenesis‐related pathways; supports prepeel conditioning [9]	Epidermal; mixed	Slow onset when used topically; early irritation requires gradual introduction and barrier monitoring [9]
Jessner's solution	Superficial	Combination keratolysis; enhances penetration of subsequent agents; mild anti‐inflammatory action [18, 19, 20]	Epidermal; mixed; selected skin of color protocols	Immediate stinging and frosting may occur; results depend strongly on operator technique and number of coats [19, 20, 27]
Lactic acid (LA)	30%–92%; superficial	Keratolysis; humectant and barrier‐supportive properties; possible tyrosinase inhibition [10, 17]	Epidermal; sensitive skin	High‐concentration protocols need standardization; evidence base remains smaller than for GA [10, 17]

### 3.2 Postinflammatory Hyperpigmentation

PIH is caused by excess epidermal and/or dermal melanin following inflammatory, infectious, traumatic, or procedural insult. It is common after acne and disproportionately affects darker phototypes [7]. Because PIH itself is a sequela of inflammation, management must balance pigment clearance against the danger of provoking further injury. This is why superficial peels are usually preferred and why disease control of the original trigger is essential before procedural treatment is intensified.

Among available peel agents, SA has one of the clearest roles in PIH, especially postacne PIH. Its lipophilicity allows penetration into sebaceous follicles, and its anti‐inflammatory and comedolytic effects help address the acne activity that often drives recurrent pigmentation [26]. Clinical studies have reported improvement in pigmentation severity and quality‐of‐life measures with serial SA peels, usually with transient dryness, erythema, or stinging rather than serious complications [27, 28].

GA is another commonly used option for PIH. Studies in darker‐skinned cohorts suggest that serial GA peels can lighten epidermal PIH and may enhance response to topical depigmenting agents [29]. Compared with SA, GA may be preferable when diffuse epidermal pigmentation predominates rather than active acne, but its success still depends on conservative escalation and adequate barrier recovery between sessions [30]. In susceptible patients, cumulative irritation may negate benefit, underscoring the importance of low starting concentrations, extended intervals, and strict sunscreen use.

Adjunctive depigmenting agents such as azelaic acid may further improve acne‐related PIH and help maintain barrier function, but they should be framed as supportive therapy rather than peel‐based intervention [31].

In practice, the best candidates for peel‐based PIH treatment are patients with stable, predominantly epidermal pigmentation, adequate control of the inciting disorder, and good adherence to photoprotection.

*Timing is critical:* peels should be deferred until active inflammation or barrier impairment is controlled. Epidermal brown macules respond better than dermal or slate‐gray lesions, and counseling should emphasize gradual improvement and maintenance.

### 3.3 Solar Lentigines and Other Localized Pigmentary Disorders

Solar lentigines are sharply demarcated macules associated with chronic ultraviolet exposure and photoaging. Because they are usually epidermal and localized, they are amenable to procedural therapy, including focal or field chemical peeling. Evidence suggests that TCA‐based focal application or field peeling may improve lentigines, especially when lesions are superficial and well defined. Response can be relatively rapid, but so can irritation and dyschromia if concentrations are too high or postprocedure photoprotection is inadequate [32].

Despite this potential, peels are not always the first choice for solar lentigines. Device‐based modalities often achieve stronger clearance for isolated lesions, especially in lighter phototypes [33, 34]. Chemical peels may therefore be most useful as lower‐cost alternatives, adjuncts when devices are unavailable, or part of broader rejuvenation regimens that address dyschromia and texture simultaneously.

Evidence for Riehl's melanosis and pigmented contact dermatitis remains limited but clinically relevant. Small case series suggest that SA‐containing protocols, often combined with topical anti‐inflammatory, depigmenting, or antioxidant therapy, may provide gradual improvement [35, 36]. The available data are preliminary and treatment is rarely standardized, but they support the concept that serial superficial peeling can contribute to multimodal management when trigger avoidance and supportive therapy are also implemented.

Periorbital hyperpigmentation is another difficult indication because causation may include melanin deposition, vascularity, laxity, tear trough shadowing, atopy, and rubbing behavior. Small studies describe benefit from GA, lactic acid, TCA, and mixed superficial peeling protocols, particularly when a pigmentary component coexists with mild textural change [37, 38]. However, the thin infraorbital skin increases the risk of irritation and inadvertent overtreatment, so peels in this region should be conservative and diagnosis driven.

Overall, outside melasma and PIH, peels should be individualized, conservative, and integrated into a diagnosis‐specific plan rather than used as a routine solution.

### 3.4 Uncommon and Site‐Specific Indications

Beyond facial melasma and PIH, chemical peels may have a limited role in uncommon or site‐specific pigmentary indications. In truncal acne and truncal acne‐associated hyperpigmentation, treatment should first control active acne, because persistent inflammation, follicular injury, and excoriation can perpetuate dyspigmentation [39].

Acne‐associated macular hyperpigmentation is common in skin of color, and expert consensus supports chemical peels as adjunctive or second‐line therapy for persistent pigmentation. SA and GA are relevant options because of their comedolytic, keratolytic, anti‐inflammatory, and epidermal‐renewal effects, but truncal and excoriated lesions require conservative protocols to avoid irritation‐induced PIH [40]. In acne excoriée, peels should be deferred until erosions, inflammation, and barrier impairment are controlled.

Minocycline‐induced facial hyperpigmentation has been described only in isolated reports and small combined‐treatment series [41]. Because pigment may be dermal‐predominant, superficial peels should be regarded as adjunctive measures, usually combined with discontinuation of minocycline, strict photoprotection, and selected light‐based therapies when appropriate.

Macular amyloidosis is a less evidence‐based indication because it is often associated with rippled hyperpigmentation, pruritus, friction, and chronic scratching; peels should be considered only cautious adjuncts to itch control, friction avoidance, topical therapy, and counseling [42].

Practical chemical peeling strategies for common pigmentary disorders are summarized in Table 2.

**TABLE 2:** Practical chemical peeling strategies for common pigmentary disorders.

Disorder	Preferred depth	Suggested agents/protocols	Combination strategy	Key cautions
Melasma	Very superficial to superficial; medium‐depth only for selected focal or refractory disease	GA 30%–70%; SA 20%–30%; LA superficial protocols; Jessner's solution; TCA 10%–20% superficial or 20%–35% with caution [8, 10, 11, 12, 13, 14, 15, 16, 17, 18, 19, 20, 21]	Strict photoprotection plus topical depigmenting therapy. Selected cases may combine peels with azelaic acid, tranexamic acid, microdermabrasion, low‐fluence IPL, or Q‐switched lasers [12, 13, 22, 23]	High relapse and PIH risk, especially in skin of color. Use priming, gradual escalation, adequate intervals, and barrier repair
Postinflammatory hyperpigmentation (PIH)	Superficial	SA 20%–30% for acne‐prone PIH; GA 30%–50% for diffuse epidermal PIH; Jessner's solution in selected protocols [7, 26, 27, 28, 29, 30]	Control acne or other inflammatory triggers first. Combine with retinoids, hydroquinone, azelaic acid, modified Kligman‐type regimens, and maintenance photoprotection [29, 30, 31, 39, 40]	Do not peel active dermatitis, excoriations, or unstable acne. Prefer low‐intensity serial treatment in darker phototypes
Solar lentigines	Superficial to medium‐depth; often focal spot treatment	Focal TCA 30%–35% for well‐demarcated lesions; GA or Jessner's solution may improve background dyschromia [32, 33, 34]	May complement cryotherapy, IPL, or lasers when broader photodamage or texture change is present [33, 34]	TCA > 35% increases risk of scarring and persistent dyschromia. Use conservative focal application and rigorous sun protection
Riehl's melanosis/pigmented contact dermatitis	Superficial; adjunctive use	SA‐containing regimens, GA or other AHAs, and composite superficial peels have been reported in small series [35, 36]	Trigger identification and avoidance are essential. Combine with sunscreen, anti‐inflammatory care, antioxidants, and depigmenting agents as appropriate [35, 36]	Evidence is limited and heterogeneous; treatment must be gradual and individualized. Ongoing allergen exposure undermines results
Periorbital hyperpigmentation	Very superficial to superficial only	Low‐concentration GA or LA; carefully selected low‐concentration TCA protocols; avoid medium or deep peels [37, 38]	Treat by subtype. Pigmentary cases may benefit from topical brighteners; mixed cases may require microneedling or other adjuncts [37, 38, 43]	Thin eyelid skin has low tolerance for irritation. Limit contact area, use longer intervals, and avoid aggressive peeling

## 4 Safety, Patient Selection, and Combination Strategies

Across all pigmentary disorders, success depends less on simply using a peel than on choosing the correct depth and protocol for the right patient. Superficial peels are the safest and most versatile for diffuse acquired hyperpigmentation because they primarily target epidermal melanin while minimizing the scarring and dyschromia risks associated with deeper injury [6, 44]. The clinician must consider phototype, barrier status, prior irritant reactions, history of PIH, active inflammation, concomitant topical use, and the patient's ability to comply with sun avoidance and maintenance therapy.

In skin of color, chemical peels should be performed conservatively because excessive inflammation or depth may induce postinflammatory hyperpigmentation, dyspigmentation, or scarring [6, 7]. For Fitzpatrick skin types III–VI, very superficial or superficial peels are preferred, while medium‐depth peels should be reserved for selected localized lesions [25]. GA and SA are generally safer for serial treatment than TCA [45]. Strict photoprotection, appropriate priming, barrier optimization, gradual escalation, and careful postpeel care are essential.

Prepeel priming is often crucial. Sunscreen is a nonnegotiable foundation of treatment. Topical depigmenting agents, retinoids, and barrier‐supportive regimens may reduce epidermal melanin load, promote more uniform penetration, and lower the chance of postprocedure complications when used appropriately before treatment [3, 24, 25]. However, overpriming with multiple irritating agents can destabilize the barrier, so regimens must be individualized.

Typical short‐term adverse effects include stinging, burning, erythema, dryness, scaling, and transient darkening [13, 24]. Most are self‐limited, but they may herald excessive cumulative irritation if session intervals are too short or patients use exfoliants at home during recovery. More serious outcomes, including persistent PIH, hypopigmentation, infection, textural alteration, and scarring, are uncommon but clinically important. These complications are usually linked to excessive depth, inappropriate self‐administration, poor selection, or inadequate postprocedure counseling [4, 24].

Patch testing is not universally required, but a conservative test approach may be useful in selected high‐risk patients, particularly those with prior peel complications or uncertain tolerance of multiple active topicals [45]. Equally important is postpeel guidance. Gentle cleansing, temporary reduction of irritating topical agents, regular emollient use, and strict ultraviolet and visible‐light protection can determine whether the procedure heals uneventfully or progresses to prolonged erythema and secondary PIH.

Combination treatment is common because peels alone seldom provide durable control of recurrent pigmentary disease. Studies suggest added benefit when peels are paired with oral tranexamic acid, microneedling, microdermabrasion, selected low‐fluence laser or light modalities, and standard depigmenting creams [13, 23, 43]. Still, the literature is heterogeneous and often limited by small samples and short follow‐up. Clinicians should therefore prioritize regimens that are reproducible, tolerable, and compatible with long‐term disease suppression.

## 5 Future Directions

Future progress in chemical peeling for pigmentary disorders will depend on improving precision, safety, and standardization. Novel formulations and delivery systems, including supramolecular SA, composite peel systems, and biostimulatory materials, are expanding the therapeutic landscape beyond traditional agents such as GA, SA, and TCA [21, 46, 47]. Rather than simply increasing peeling strength, these newer approaches aim to improve tolerability, depth control, and biocompatibility, particularly in patients with darker skin phototypes who are more vulnerable to treatment‐induced dyschromia.

At the same time, peeling strategies are moving toward a more personalized model. Melasma, postinflammatory hyperpigmentation, Riehl's melanosis, and periorbital hyperpigmentation differ substantially in pathogenesis, pigment depth, and recurrence risk; accordingly, optimal treatment requires individualized selection of peel agent, concentration, depth, and treatment interval. Future protocols should increasingly incorporate objective assessment tools, including Wood's lamp examination and other pigment‐localization methods, to better align treatment intensity with disease subtype and skin phototype.

Another priority is the standardization of combination therapy. Available evidence suggests that chemical peels are most effective as adjunctive interventions used alongside topical depigmenting agents (e.g., hydroquinone, tretinoin, azelaic acid), systemic therapies (e.g., tranexamic acid), or selected energy‐based procedures (e.g., low‐fluence IPL, Q‐switched lasers) [22, 23, 48]. However, substantial heterogeneity remains in treatment sequencing, interval design, and cumulative exposure. Consensus‐based multimodal frameworks are needed to improve reproducibility and facilitate wider clinical adoption.

Finally, the evidence base remains limited by small sample sizes, single‐center designs, short follow‐up, and inconsistent outcome measures. Well‐designed multicenter randomized controlled trials with longer follow‐up are needed to clarify comparative efficacy, durability, and safety across different peeling agents, protocols, and pigmentary subtypes.

## 6 Conclusion

Chemical peeling remains an important component of the therapeutic armamentarium for pigmentary skin disorders. Its role has evolved beyond simple exfoliation to a broader modality that promotes epidermal turnover, facilitates pigment dispersion, enhances penetration of topical therapies, and may contribute to superficial remodeling and inflammatory control. Current evidence supports superficial and very superficial peels as the most practical and safest options for common disorders such as melasma, postinflammatory hyperpigmentation, and periorbital hyperpigmentation, whereas selected medium‐depth peels may be useful for solar lentigines and certain refractory lesions in carefully chosen patients.

Clinical success depends not only on the peeling agent itself, but also on appropriate patient selection, priming, protocol design, and postprocedure care. This is particularly important in darker skin phototypes, in whom overly aggressive treatment may worsen dyspigmentation. Overall, chemical peels should be regarded not as stand‐alone cures, but as efficacy‐enhancing components of individualized, multimodal treatment strategies. With continued advances in formulation science, disease stratification, and protocol standardization, chemical peeling is likely to become an increasingly precise, safer, and more personalized option in cosmetic dermatology.

## Author Contributions

Y.L. and W.X. contributed to the conception and design of the review. Y.L. performed the literature search and drafted the manuscript. W.X. critically revised the manuscript. All authors approved the final version of the manuscript.

## Funding

This work was supported by Construction Fund of Key Medical Disciplines of Hangzhou (2025HZGF07), Hangzhou Key Scientific Research Program Projects (2025SZD2B04), and Zhejiang Province Health Industry Science and Technology Planning Project (2025HY0696).

## Ethics Statement

The authors have nothing to report.

## Consent

The authors have nothing to report.

## Conflicts of Interest

The authors declare no conflicts of interest.

## Data Availability

Data sharing not applicable to this article as no datasets were generated or analysed during the current study.
